# Season of birth has no effect on symptoms of depression and anxiety in older adults

**DOI:** 10.1038/s41598-022-10892-8

**Published:** 2022-04-26

**Authors:** Zsófia Csajbók, Anna Kagstrom, Pavla Cermakova

**Affiliations:** 1 Second Faculty of Medicine, Charles University Prague, V Úvalu 84, 15006 Prague 5, Czechia; 2 Faculty of Humanities, Charles University Prague, Prague, Czechia; 3 National Institute of Mental Health, Klecany, Czechia

**Keywords:** Risk factors, Psychology

## Abstract

There remains a lack of conclusive evidence as to the merit of season of birth as a predictor of mental illness across contexts. We studied 72,370 individuals (55% women; mean age 66) from the Survey on Health, Ageing and Retirement in Europe. Depressive symptoms were assessed with EURO-D scale and symptoms of anxiety with modified Beck Anxiety Inventory. Multilevel modeling was used to assess the association of season of birth as well as month of birth with symptoms of depression and anxiety, by sex and region. Adjusting for sex and age, month of birth explained only 0.01% to 0.07% of anxiety and depressive symptoms with non-significant improvement in the overall models; using season of birth instead of month of birth added 0.00% to 0.04% of explained variance. When stratified by sex and European region, age explained 0.23% to 5.19% of anxiety and depressive symptoms; the addition of month of birth or season of birth improved the models by negligible amount. Season of birth and month of birth are not reliable predictors of anxiety and depression across the life course.

## Introduction

The demographically ageing population brings several challenges with respect to the health of older adults. At least one third of older adults experience a mental illness, most commonly depression and anxiety^1^. The burden of the onset and severity of depression and anxiety could be largely mitigated by reducing risk factors for their development, especially if targeted early in life^2,3^. The state of one’s mental health across the life-course is determined by a range of contributing biological, psychological, social, and developmental factors, which can influence disease risk. Understanding underlying mechanisms of risk and protective factors across the life-course and variables, such as the timing of exposure and their function on brain development is foundational to assessing risk and providing targeted interventions to those most vulnerable to depression and anxiety later in life^4,5^. The nature and timing of environmental contributions together with sensitive periods, during which an individual is at greatest risk of damage if exposed, can set off a chain of adverse events leading to depression and anxiety^6^.

Season of birth (SOB) has been largely studied in the context of the life-course perspective on mental health. Within mental disorders, the most pervasive studies widely cited include those indicating that SOB is associated with increased risk of schizophrenia and bipolar disorder^7,8^, schizo-affective disorder, and major depression^8^. Some studies have also found SOB to be associated with anorexia nervosa^9,10^, personality disorder^11,12^, panic disorder^13^, autism^14,15^, risk of suicide^16,17^ and cognitive functions^18^. However, the evidence-base is greatly biased by confounding factors^19^, especially socioeconomic position, and many studies show a relatively low value of SOB as a predictor of mental illness, including for example a study conducted in the Netherlands on SOB as a predictor of bipolar episodes^20^. A recent systematic review reported a population attributable risk of only 3.3% for being born during winter and spring months and the development of schizophrenia^19^.

SOB as a predictor of mental health has proven variation in observed results according to the time and place measured. Existing studies due to their lack of generalisability require historical context for interpretation of findings and unearthing of underlying explanatory mechanisms^19^. Some explanatory theories are rooted in genetic influences, for example, the theory that the procreation habits of parents with genetic risk factors inherently differ in seasonality from parents without hereditary risks^21^. However, the most widely accepted explanations are those surrounding mechanisms, which are greatly affected by social determinants of health: nutrition and disease exposure. Generally, the two pervasive mechanisms, which can explain the variation of results found in studies examining SOB and mental health are seasonal variation in nutrient supply, and seasonal variation in disease exposure^22^. While these two mechanisms differ, they reflect the theory that some in-utero exposure or deficiency impacts fetal development, for example exposure to infectious diseases, such as the flu^23^, which is highly correlated with specific seasons^24^. However, it is likely that for individuals born into socioeconomically advantaged contexts, SOB may pose no or little effect on their later-life health outcomes as sufficient socioeconomic recourses can address and eliminate the underlying health mechanisms associated with seasonal variation.

The source of the association between SOB and health over the life course has been linked to adaptive developmental plasticity processes which are influenced by environmental factors^25^. Differences are in part explained by the effect of SOB on the postnatal development of circadian rhythm functioning, and although most supporting hypotheses come from rodent studies, SOB and associations with offspring’s long-term health and welfare have been shown to have roots both via epigenetic effects and postnatal programming. These factors, when facing different light environments, pose different effects on offspring’s brain, physiology, and behavior^26^. Human studies suggest that melatonin signaling during the perinatal photoperiod interacts with serotonergic pathways impacting emotional behaviors in offspring^27^ as well as prenatal programming, which affects circadian and limbic systems^28^.

In the context of the shifting burden of disease and improved socioeconomic conditions in Europe, the relationship between early life factors and mental health risk in older age is far less explored compared to physical health. SOB and health implications have been previously studied examining variations in health outcomes in older age according to national and geographical contexts^29^, however, to our knowledge no research has explored the effects of SOB on symptoms of depression and anxiety in older adults using a large-scale multi-national sample. In the present study, we aimed to investigate the association of SOB with symptoms of depression and anxiety in a large study of older Europeans. We hypothesize that SOB poses only little effect on their later-life mental health.

## Results

**Figure 1:**
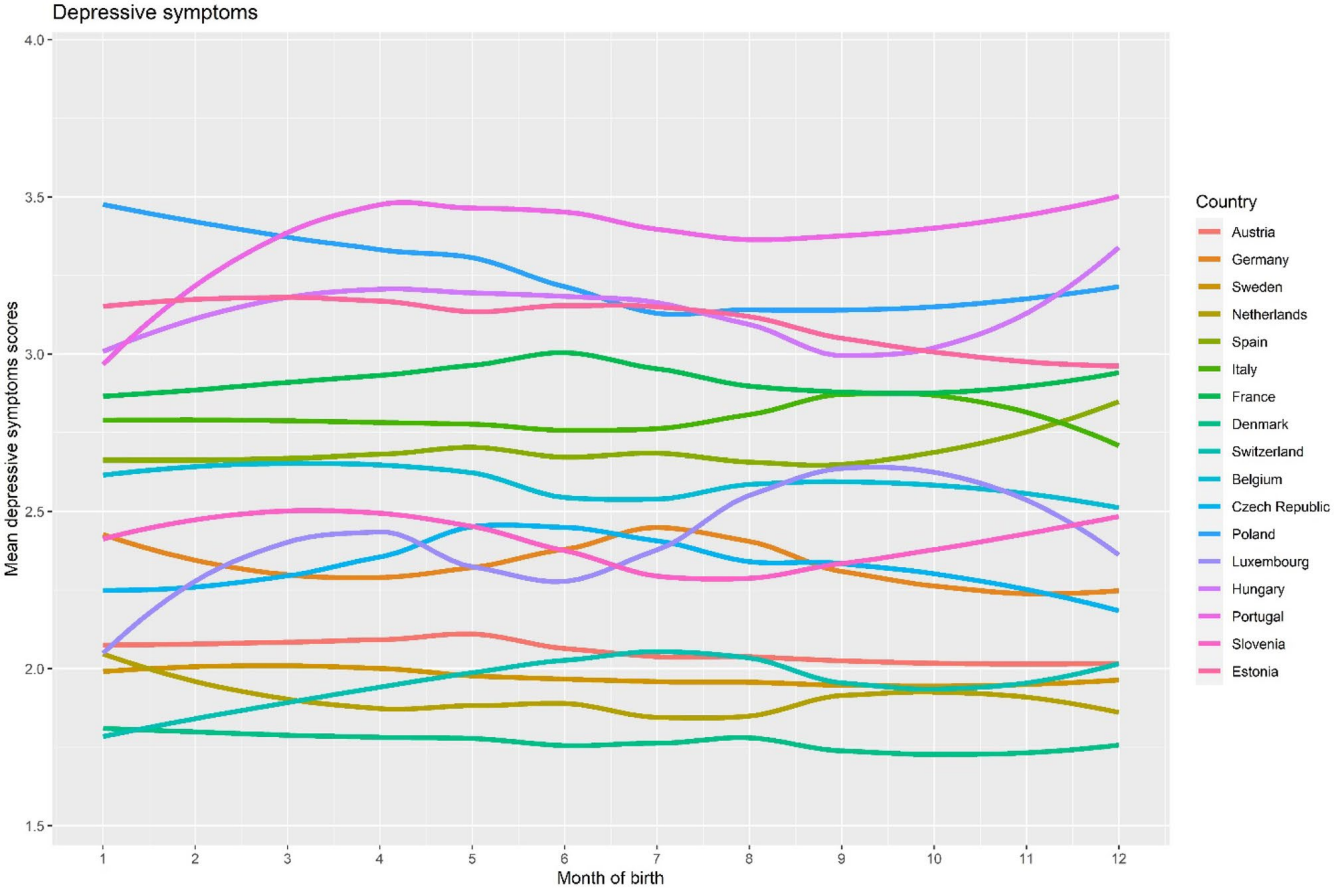
Mean depressive symptoms by country and month of birth (depressive symptoms scores ranged between 0 to 12; smoothing splines were fitted against mean scores).

**Figure 2:**
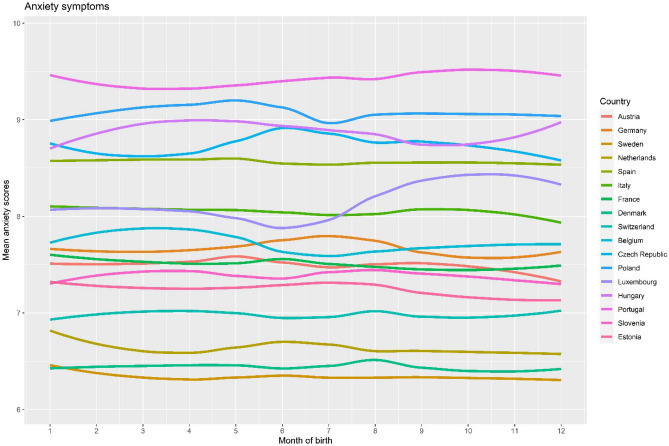
Mean anxiety symptoms by country and month of birth (anxiety scores ranged between 5 to 20; smoothing splines were fitted against mean scores).

We studied 72,370 people (55% women, mean age 66 ± 10 years). Table 1 presents distributions of age, sex, and depressive and anxiety symptoms across SOB. Age showed a significant difference across SOB, but with a negligible effect size. Sex, and depressive and anxiety symptoms did not differ across the SOB. Figures 1 and 2 present mean anxiety and depressive symptoms scores plotted across month of birth (MOB) for each country using smoothing splines. Visual inspection of the figures showed us that although some fluctuation can be seen in depressive symptoms in certain countries (e.g., Hungary, Portugal), and in anxiety symptoms (e.g., Czech Republic, Luxembourg), and overall, countries differ in their mean depressive and anxiety symptoms, no visible systematic pattern can be found for the association of MOB with depressive/anxiety symptoms.

**Table 1:** Characteristics of participants by season of birth.

	Spring (n = 19,239)	Summer (n = 17,804)	Autumn (n = 17,383)	Winter (n = 17,944)	p value	Effect size (η_p_^2^ or V)
Age (mean ± SD)	65.59 (9.88)	65.51 (9.91)	65.50 (9.98)	65.99 (10.04)	< .001	< .001
Women (n, %)	10,554 (54.9%)	9915 (55.7%)	9580 (55.1%)	9835 (54.8%)	.312	.007
Depressive symptoms (mean ± SD)	2.51 (2.30)	2.49 (2.29)	2.46 (2.25)	2.48 (2.27)	.139	< .001
Anxiety symptoms (mean ± SD)	7.70 (2.85)	7.68 (2.83)	7.68 (2.85)	7.71 (2.86)	.648	< .001

The unconditional model suggested some variance attributable to country differences (i.e., random intercept of country) with intraclass correlations between 0.047 to 0.113 (Table 2). Adjusting for sex and age explained between 1.36% to 6.33% of the residual within-country variance of anxiety and depressive symptoms on the total sample and, also stratified by age groups. Adding these fixed effects yielded significant improvement in the log likelihood of the models. Entering MOB in the models explained between 0.01 to 0.07% of the residual variance of anxiety and depressive symptoms with non-significant improvement in the overall models. Using SOB instead of MOB yielded similarly low added explained variance of the models between 0.00 to 0.04% (Table 3).

**Table 2:** Multilevel models predicting anxiety and depressive symptoms with months of birth adjusted for sex and age.

	Predictors (fixed effects)^a^	Anxiety	Anxiety (age < 65)	Anxiety (age ≥ 65)	Depressive symptoms	Depressive symptoms (age < 65)	Depressive symptoms (age ≥ 65)
ICC (Model 0)		0.092	0.077	0.113	0.055	0.047	0.075
Model 1	Sex (men)	−0.696***	−0.606***	−0.752***	−0.828***	−0.753***	−0.872***
	Age	0.037***	−0.002	0.069***	0.026***	−0.009	0.054***
Explained variance^b^		3.40%***	1.36%***	4.56%***	4.67%***	3.04%***	6.33%***
Model 2	Sex (men)	−0.696***	−0.605***	−0.752***	−0.828***	−0.753***	−0.873***
	Age	0.037***	−0.002	0.069***	0.026***	−0.009	0.054***
	February	−0.019	0.011	−0.047	−0.058	0.001	−0.115^+^
	March	−0.065	0.028	−0.155*	0.002	0.058	−0.056
	April	0.016	0.061	−0.015	0.039	0.067	0.020
	May	0.057	0.138^+^	−0.020	0.063	0.106	0.020
	June	−0.001	0.091	−0.104*	−0.012	−0.034	0.001
	July	−0.023	0.040	−0.080	−0.002	0.018	−0.017
	August	0.007	0.042	−0.031	0.031	0.069	−0.012
	September	−0.010	0.060	−0.063	−0.049	−0.020	−0.066
	October	−0.021	0.033	−0.072	−0.031	−0.040	−0.019
	November	−0.104**	−0.045	−0.176**	−0.020	−0.028	−0.026
	December	−0.022	0.033	−0.078	−0.005	0.001	−0.015
Added explained variance^c^		0.01%	0.03%	0.04%	0.02%^+^	0.07%	0.04%

ICC = intraclass correlation, i.e., variance attributable to cluster (country) differences.

^a^ The models are clustered across countries.

^b^ Added explained within country residual variance of the fixed effect of sex and age in comparison to Model 0. Model 0 is the unconditional model splitting the variance of the dependent variable into within cluster and between cluster parts.

^c^ Added explained within country residual variance of the fixed effects of birth months in comparison to Model 1.

^+^p < 0.1; *p < 0.05; **p < 0.01; ***p < 0.001.

**Table 3:** Multilevel models predicting anxiety and depressive symptoms with seasons of birth adjusted for sex and age.

	Predictors (fixed effects)^a^	Anxiety	Anxiety (age < 65)	Anxiety (age ≥ 65)	Depressive symptoms	Depressive symptoms (age < 65)	Depressive symptoms (age ≥ 65)
Model 1	Sex (men)	−0.696***	−0.606***	−0.752***	−0.828***	−0.753***	−0.872***
	Age	0.037***	−0.002	0.069***	0.026***	−0.009	0.054***
Explained variance^b^		3.40%***	1.36%***	4.56%***	4.67%***	3.04%***	6.33%***
Model 2	Sex (men)	−0.696***	−0.606***	−0.752***	−0.828***	−0.753***	−0.872***
	Age	0.037***	−0.002	0.069***	0.026***	−0.009	0.054***
	Summer	−0.007	−0.019	−0.007	−0.029	−0.060*	−0.003
	Autumn	−0.045	−0.059	−0.036	−0.068**	−0.107**	−0.031
	Winter	−0.015	−0.062*	0.026	−0.055**	−0.077*	−0.037
Added explained variance^c^		0.00%	0.02%	0.01%	0.02%*	0.04%*	0.02%

Model 0 and Model 1 are the same as in the birth month prediction models.

^a^The models are clustered across countries.

^b^Added explained within country residual variance of the fixed effect of sex and age in comparison to Model 0. Model 0 is the unconditional model splitting the variance of the dependent variable into within cluster and between cluster parts.

^c^Added explained within country residual variance of the fixed effects of birth months in comparison to Model 1.

*p < 0.05; **p < 0.01; ***p < 0.001.

When comparing younger and older cohorts (below and above 65 years of age), the additional variance of anxiety explained by MOB did not meaningfully differ across age groups (0.03% below 65 years and 0.04% above 65 years). Similarly, MOB did not explain better depressive symptoms in the older than in the younger cohort (0.07% below 65 years and 0.04% aged 65 years and more). When predicting by SOB, the added explained variances were virtually the same in both age groups (anxiety symptoms: 0.02% in the younger, and 0.01% in the older cohort; depressive symptoms: 0.04% in the younger and 0.02% in the older cohort).

The effect of MOB was also tested on the sample stratified by sex and European region (Table 4). While age explained between 0.23 to 5.19% of anxiety and depressive symptoms, MOB introduced only between 0.03 to 0.44% improvement in the regression models. Although the effects of MOB were not statistically significant, the strongest were found among Scandinavian men and women, and weakest among Western Europeans.

**Table 4:** Variance explained by Age (Model 1) and Month of birth added (Model 2) across sex and European regions.

			Anxiety (%)	Depressive symptoms (%)
Western Europe & Women	Model 1 (Age)	Explained variance^a^	1.05***	0.50***
	Model 2 (Age + Birth month)	Added explained variance^b^	0.06	0.06
Scandinavia & Women	Model 1 (Age)	Explained variance	0.23**	0.29***
	Model 2 (Age + Birth month)	Added explained variance	0.26	0.10
Southern Europe & Women	Model 1 (Age)	Explained variance	5.19***	4.77***
	Model 2 (Age + Birth month)	Added explained variance	0.11	0.07
Central and Eastern Europe & Women	Model 1 (Age)	Explained variance	2.35***	2.44***
	Model 2 (Age + Birth month)	Added explained variance	0.13	0.11
Western Europe & Men	Model 1 (Age)	Explained variance	0.54***	0.26***
	Model 2 (Age + Birth month)	Added explained variance	0.03	0.04
Scandinavia & Men	Model 1 (Age)	Explained variance	0.50***	0.45***
	Model 2 (Age + Birth month)	Added explained variance	0.44^+^	0.19
Southern Europe & Men	Model 1 (Age)	Explained variance	3.73***	3.65***
	Model 2 (Age + Birth month)	Added explained variance	0.13	0.16
Central and Eastern Europe & Men	Model 1 (Age)	Explained variance	2.02***	1.29***
	Model 2 (Age + Birth month)	Added explained variance	0.10	0.13

^a^Explained variance is significant if the F statistic is significant of Model 1.

^b^Added explained variance is significant if the F change is significant between Model 1 and Model 2.

^+^p < .1; **p < 0.01; ***p < 0.001.

## Discussion

In the present study, capitalizing on a well-characterized sample of more than 70,000 middle-aged and older individuals, we observed that SOB had virtually no effect on symptoms of depression and anxiety. While having been a topic of study for decades, the relationship between SOB and risk for depression and anxiety over the life-course has previously lacked large-scale generalizable evidence. Previous studies have suggested an effect of SOB on several brain disorders, with the most consistent association found for being born in winter and increased risk for schizophrenia^8,30^. This association was primarily explained by varying exposures during the perinatal period related to viral infections, climate influences, differences in sunlight or levels of vitamin D^31–38^. Nutritional deficiencies also are at the basis of explanatory hypothesis for SOB as a predictor of mental health over the life course. The effect of the variety of nutritional intake available^38,39^ on pregnant women and the fetus^40^ is a well-documented health determinant over the life course. Early nutritional exposures have been found to be independently related to the risk for behavioral and emotional problems in children ^40^, and more generally, nutrition is a well-established and fundamental health predictor across the life course. Improvements in both knowledge about specific nutrients related to disease risk and protection as well as access to food supply over time have led to improved health in populations^40^, which may also explain the heterogeneity of past studies and the lack of a current significant association between SOB and mental illness in the present study.

Only a few of previous studies assessed the association of SOB with depressive and anxiety symptoms, and, to our knowledge, none have been conducted on large-scale samples with variations in population nations and latitude. A study in Korea examining SOB and major depressive disorders found that patients born in spring/summer were on average younger at the onset of the first depressive episode and experienced more severe clinical symptoms than those born in autumn/winter^41^. Another study from China found an effect of SOB in people with obsessive–compulsive disorder, especially for men^42^. The lack of clarity in the field has led to skepticism on SOB as a meaningful health determinant, and historically the research focused on SOB and mental health has dwindled over time^19^.

Some previous studies suggested evidence of sex and regional specific associations of SOB with ageing and several characteristics, such as personality and sleeping habits^35,43^. Abeliansky et al. found that older men, particularly in Northern European countries, age faster when they were born in spring. On the contrary, SOB did not play a role for health in older age in Southern countries. A different amount of light during the year and availability of fruit was suggested to be the underlying mechanism^29^. However, in our study, we found only small and non-significant regional effects, albeit the strongest among Scandinavian men and women, and weakest among Western Europeans. These non-significant effects could be explained by the previously discussed adaptive developmental plasticity associated with different light environment^25,26^.

A recent US study, which re-visited the relationship on SOB and depression in adulthood, aimed to explore SOB as a predictor of depression in adulthood^19^. Through analyzing the effects of birth month interacting with birth year on depression in adulthood, the author finds significant effects with gradual weakening of the relationship over time. The results from the present study and the US study lead to overwhelming evidence that SOB is of diminishing relevance as a reliable predictor of mental health problems over time and across contexts. These results can expand the process of scientific advances leading to the uncovering of nutritional deficiencies, which over time have been decreased, resulting in overall improvements in population health. Correlation is not causation and past studies while finding correlation are less and less supported as the mitigating health influences of this correlation are solved via health interventions prompted by understanding of the underlying mechanisms of risk factors for mental illnesses.

This study has several limitations. First, participants of SHARE are not fully representative to the general population as people who are more educated and more interested in their health tend to participate in such surveys. Therefore, this sample may be mentally healthier than general population, which could possibly lead to the dilution of the association of SOB with symptoms of depression and anxiety. Second, we could assess only symptoms of depression and anxiety and not clinical depression or anxiety disorders, therefore, clinical significance of our findings is unclear. This was done because our research question was driven from a public health perspective, and we aimed to determine the effect of SOB on mental health in the whole population. However, we cannot rule out that there is, in fact, an association of SOB with clinical depression or anxiety disorders. Third, while we found that the Scandinavian population had the highest variance explained of all regions, it is difficult to discern the source of this observed variance. It is possible that the source of SOB and the explained variance in Northern Europe are in fact related to the higher latitudes and photoperiodic differences between the months, as is suggested in an older study on Swedish men^44^. However, the model including MOB offered no significant change to the explanation of the association between mental illness risk, therefore, it is likely that additional factors and mediators, which were not measured, could explain these findings. For example, we did not account for additional factors and mediators, which are known to impact mental health outcomes, e.g., nutritional deficiencies. However, as we did not detect any association between SOB with symptoms of depression and anxiety, adding more covariates would not impact the main results of the study. Fourth, the instruments used in this study to measure depressive and anxiety symptoms were not validated for each country. Future research could overcome these limitations by choosing a sample of participants that is representative towards the general population, has been assessed also clinically for depression and anxiety disorders and uses instruments validated for each country.

Taking into consideration the strong evidence-base indicating nutrition and disease exposure as predictors of health over the life-course, and the present results indicating no association between SOB and symptoms of depression and anxiety, it seems that SOB as a predictor of mental health is insufficient. Historically, when SOB was found significantly associated with mental illness, it was likely successfully acting as a proxy indicator for an unaccounted-for mediating mechanism. The attenuating trajectory of the relationship between SOB and mental health highlights the importance of unearthing driving mechanisms of socio-environmental determinants of health and re-evaluating and interpreting past findings in light of advances in scientific discoveries as a step towards making meaningful interpretations of findings. Future research should aim to unearth underlying mechanisms associated in contexts where SOB has been found to be associated with mental illnesses, further exploring associations between biological risk factors, which result from environmental conditions such as SOB as direct predictors of mental health across the life-course.

## Methods

### Source of data

Data for the analysis were used from a prospective, multidisciplinary and multi-centre study Survey on Health, Ageing and Retirement in Europe (SHARE) that aims to study population ageing across Europe^1,45^. Data on health, social network and economic conditions are collected from community-dwelling individuals (aged at least 50 + years old) and their partners, irrespective of age, using computer-assisted personal interviewing (CAPI), as previously described in detail^45^. For this study, data was available for 27 European countries and Israel from 7 waves: wave 1 was conducted in 2004, followed by wave 2 in 2006/2007, wave 3 in 2008/2009, wave 4 in 2011/2012, wave 5 in 2013, wave 6 in 2015 and wave 7 in 2017.

### Season of birth (SOB)

Information about the date of birth was acquired directly from participants as a part of CAPI. Our sample was then divided into 4 groups based on the month of birth (MOB): winter (individuals born in December, January, February), spring (born in March, April, May), summer (born in June, July, August) and fall (born in September, October, November). We use both MOB as well as SOB in our analyses.

### Depressive symptoms

Depressive symptoms were assessed using EURO-D scale, which was administered by trained interviewers as a part of CAPI. The EURO-D scale was developed and validated to compare symptoms of depression in older adults across Europe^46^. The 12 EURO-D items (depressed mood, pessimism, wishing death, guilt, sleep, interest, irritability, appetite, fatigue, concentration, enjoyment and tearfulness) are scored 0 (when symptom is not present) or 1 (when symptom is present). This generates a scale ranging from 0 to 12, with higher scores indicating a greater severity of depressive symptoms.

### Anxiety symptoms

Anxiety symptoms were assessed using a five-item scale based on a modified Beck Anxiety Inventory^47^. One item is about psychological (fear of the worst happening), two items about physiological (feeling my hands trembling, feeling faint) and two items about cognitive symptoms (being nervous, having a fear of dying) associated with anxiety. The questions are answered on a four-point Likert scale (“never”, “hardly ever”, “some of the time”, “most of the time”). A sum of these five items creates the final anxiety scale, with higher score indicating greater anxiety.

### Analytical sample

As anxiety symptoms were assessed only in waves 4 and 5, we restricted the sample to individuals who participated in waves 4 or 5 and have at least one measure of depressive as well as anxiety symptoms. From 139,556 individuals who had at least one interview in SHARE, 85,334 people took part in waves 4 or 5. From them, we excluded participants from Israel (n = 2599) in order to focus only on Europeans, individuals with missing data on date of birth (n = 13), those not born in the country of interview (n = 6988), individuals with missing data on depressive (n = 2343) and anxiety (n = 65) symptoms in both waves, those who did not have data on both measures in the same wave (n = 119) and those younger than 50 years (n = 837), leaving 72,370 people in the final analytical sample. The sample thus included participants from Western Europe: Austria (6.7%), Germany (7.5%), Netherlands (6.0%), France (7.7%), Switzerland (4.5%), Belgium (8.4%), Luxembourg (1.4%); Scandinavia: Denmark (5.7%), Sweden (6.4%); Southern Europe: Spain (9.1%), Italy (7.5%), Portugal (2.5); and Central and Eastern Europe: Czech Republic (8.9%), Poland (2.3%), Hungary (4.0%), Slovenia (4.4%), and Estonia (6.9%). The age of the sample ranged between 50 to 111 years at the time of the interview, meaning that the year of birth ranged between 1900–1963. We split the sample at the age of 65 years for further analysis in order the inspect the cohort effect of being born during the world wars (49.6%) or after the wars (50.4%).

### Statistical analysis

Data of depressive symptoms and anxiety was used from the wave it was first available, therefore, we conducted our analysis cross-sectionally. Descriptive data is presented as mean ± standard deviation (SD), or frequency (n, %), where appropriate. First, univariate analysis was performed by comparing age and the level of depressive and anxiety symptoms between SOB using one-way analysis of variance (ANOVA). The difference in proportion of women across SOB was tested using Chi-square test. Second, multilevel modelling was applied to study the association of SOB as well as MOB with the level of depressive and anxiety symptoms. Anxiety and depressive symptoms were predicted in three consecutive two-level random intercept models using Maximum Likelihood Robust (MLR) estimator. In the unconditional Model 0, the variance of depressive and anxiety symptoms was separated into within country and between country variances using country of birth as a clustering variable to control for the differing latitude across countries. In Model 1, the fixed effects of control variables sex (female = 0, male = 1) and age were entered to the model. Model 2 introduced the fixed effects of SOB and MOB in separate models. Models 0 and 1 as well as Models 1 and 2 were compared with Chi-square comparison tests adjusted for the MLR scaling correction factor following the recommended procedure^48^. The explained variance of the fixed effects was expressed by the relative decrease of the within-country variances. Third, we explored whether the association of SOB/MOB with depressive/anxiety symptoms differs for people younger than 65 and older than 65 years by repeating the models while stratifying on age group. Lastly, we stratified the sample across sex and European regions and performed linear regression predicting depressive and anxiety symptoms with MOB or SOB. Country of birth was not controlled for here, as the variation within European regions (due to low number of countries within each region) did not permit to estimate random effects for country. The analyses were performed in Mplus (version 8.4) and the figures were created in R (version 4.1.0).

### Ethical issues

This study was carried out in accordance with the Declaration of Helsinki. SHARE has been repeatedly reviewed and approved by the Ethics Committee of the University of Mannheim. All participants provided a written informed consent. Data were pseudo-anonymized and participants were informed about the storage and use of the data and their right to withdraw consent. The present analysis was approved by the Ethics Committee of the National Institute of Mental Health, Czech Republic.

## Supplementary Information

Supplementary Tables.

## Data Availability

Access to the SHARE data is provided free of charge on the basis of a release policy that gives quick and convenient access to all scientific users worldwide after individual registration. All details about the application and registration process can be found on this website: share-project.org. The study protocol and syntax of the statistical analysis will be shared upon request from the corresponding author of this study.
