# Supplementary material for: Season of birth has no effect on symptoms of depression and anxiety in older adults
Source: Sci Rep. 2022 Apr 26;12:6823. doi: 10.1038/s41598-022-10892-8 (PMC9042812; doi:10.1038/s41598-022-10892-8)
Supplement: Supplementary file 1 — Supplementary Tables. [file 41598_2022_10892_MOESM1_ESM.docx]

| **Supplementary Table 1.**  Detailed results of the multilevel models predicting Anxiety and Depressive symptoms with birth month adjusted for sex and age | | | | | | | |
| --- | --- | --- | --- | --- | --- | --- | --- |
| **Model** |  | **Anxiety** | **Anxiety (age < 65)** | **Anxiety (age ≥ 65)** | **Depressive symptoms (continuous)** | **Depressive symptoms (continuous, age < 65)** | **Depressive symptoms (continuous, age ≥ 65)** |
| ICC (i.e., variance attributable to group differences) |  | 0.098 | 0.086 | 0.116 | 0.046 | 0.035 | 0.065 |
| Unconditional model (Model 0) | Unexplained within country variance | 7.434*** | 6.624*** | 8.065*** | 4.994*** | 4.666*** | 5.228*** |
|  | Unexplained between country variance | 0.807*** | 0.622*** | 1.061*** | 0.242*** | 0.171*** | 0.365*** |
|  | Log-likelihood | -175328.025 | -86235.224 | -88494.077 | -160928.414 | -79839.249 | -80704.535 |
|  | Scaling correction factor for MLR | 63.6969 | 24.9834 | 40.5199 | 43.7975 | 13.7743 | 30.6770 |
|  | N of free parameters | 3 | 3 | 3 | 3 | 3 | 3 |
| Fixed effects of controls (sex and age) added (Model 1) | Unexplained within country variance | 7.181*** | 6.534*** | 7.697*** | 4.761*** | 4.524*** | 4.897*** |
|  | Unexplained between country variance | 0.806*** | 0.618*** | 1.061*** | 0.236*** | 0.170*** | 0.350*** |
|  | Log-likelihood | -174078.286 | -85983.751 | -87655.826 | -159196.277 | -79274.551 | -79529.111 |
|  | Scaling correction factor for MLR | 43.3747 | 16.0046 | 25.9810 | 31.5804 | 9.6782 | 19.2986 |
|  | N of free parameters | 5 | 5 | 5 | 5 | 5 | 5 |
| Comparison of Model 1 with Model 0 | Scaling corrected chi-square difference test based on loglikelihood values (X^2^(Δdf))^a^ | 193.887(2)*** | 198.291(2)*** | 401.784(2)*** | 261.361(2)*** | 319.576(2)*** | 1053.719(2)*** |
|  | Added explained within country residual variance (compared to Model 0) | 3.40%*** | 1.36%*** | 4.56%*** | 4.67%*** | 3.04%*** | 6.33%*** |
| Fixed effect of birth month added (Model 2) | Unexplained within country variance | 7.180*** | 6.532*** | 7.694*** | 4.760*** | 4.521*** | 4.895*** |
|  | Unexplained between country variance | 0.807*** | 0.619*** | 1.062*** | 0.237*** | 0.170*** | 0.350*** |
|  | Log-likelihood | -174070.935 | -85978.395 | -87649.477 | -159187.544 | -79265.940 | -79523.934 |
|  | Scaling correction factor for MLR | 14.1639 | 5.6991 | 8.7459 | 10.4994 | 3.7171 | 6.7043 |
|  | N of free parameters | 16 | 16 | 16 | 16 | 16 | 16 |
| Comparison of Model 2 with Model 1 | Scaling corrected chi-square difference test based on loglikelihood values (X^2^(Δdf)) | 16.589(11) | 10.556(11) | 13.927(11) | 19.044(11)+ | 17.094(11) | 10.569(11) |
|  | Added explained within country residual variance (compared to Model 1) | 0.01% | 0.03% | 0.04% | 0.02%+ | 0.07% | 0.04% |
| Note: ICC = intraclass correlation. MLR = Maximum Likelihood Robust estimator. df = degree of freedom.  ^a^ As MLR estimator was used, the Chi-square comparison test was adjusted following the recommended procedure for MLR (Satorra, 2000).  + p < 0.1. * p < 0.05. ** p < 0.01. *** p < 0.001. | | | | | | | |

| **Supplementary Table 2.**  Detailed results of the multilevel models predicting Anxiety and Depressive symptoms with birth season adjusted for sex and age | | | | | | | |
| --- | --- | --- | --- | --- | --- | --- | --- |
| **Model** |  | **Anxiety** | **Anxiety (age < 65)** | **Anxiety (age ≥ 65)** | **Depressive symptoms (continuous)** | **Depressive symptoms (continuous, age < 65)** | **Depressive symptoms (continuous, age ≥ 65)** |
| N |  | 81256 | 41037 | 40219 | 61724 | 35126 | 26598 |
| Fixed effect of birth season added (Model 2) | Unexplained within country variance | 7.181*** | 6.533*** | 7.696*** | 4.760*** | 4.522*** | 4.896*** |
|  | Unexplained between country variance | 0.806*** | 0.619*** | 1.061*** | 0.237*** | 0.170*** | 0.350*** |
|  | Log-likelihood | -174076.827 | -85981.791 | -87654.706 | -159191.030 | -79268.264 | -79528.100 |
|  | Scaling correction factor for MLR^a^ | 27.4952 | 10.4906 | 16.5606 | 20.1847 | 6.4793 | 12.4234 |
|  | N of free parameters | 8 | 8 | 8 | 8 | 8 | 8 |
| Comparison of Model 2 with Model 1 | Scaling corrected chi-square difference test based on loglikelihood values (X^2^(Δdf)) | 2.835(3) | 3.014(3) | 2.605(3) | 8.805(3)* | 10.955(3)* | 2.096(3) |
|  | Added explained within country residual variance (compared to Model 1) | 0.00% | 0.02% | 0.01% | 0.02%* | 0.04%* | 0.02% |
| Note: **Model 0 and Model 1 are the same as in Supplementary Table 1.** MLR = Maximum Likelihood Robust estimator. df = degree of freedom.  ^a^ As MLR estimator was used, the Chi-square comparison test was adjusted following the recommended procedure for MLR (Satorra, 2000).  * p < 0.05. | | | | | | | |

**Reference:**

Satorra, A. (2000). Scaled and adjusted restricted tests in multi-sample analysis of moment structures. In R. D. H. Heijmans, D. S. G. Pollock, & A. Satorra (Eds.), *Innovations in multivariate statistical analysis. A Festschrift for Heinz Neudecker* (pp. 233–247). London: Kluwer Academic Publishers.
